# *TERT* promoter hotspot mutations are recurrent in myxoid liposarcomas but rare in other soft tissue sarcoma entities

**DOI:** 10.1186/1756-9966-33-33

**Published:** 2014-04-11

**Authors:** Christian Koelsche, Marcus Renner, Wolfgang Hartmann, Regine Brandt, Burkhard Lehner, Nina Waldburger, Ingo Alldinger, Thomas Schmitt, Gerlinde Egerer, Roland Penzel, Eva Wardelmann, Peter Schirmacher, Andreas von Deimling, Gunhild Mechtersheimer

**Affiliations:** 1 Department of Neuropathology, Institute of Pathology, University Hospital, Heidelberg, Germany; 2 German Cancer Consortium (DKTK), CCU Neuropathology, German Cancer Research Center (DKFZ), Heidelberg, Germany; 3 Department of General Pathology, Institute of Pathology, University Hospital, Im Neuenheimer Feld 224, Heidelberg D-69120, Germany; 4 Gerhard-Domagk Institute of Pathology, University Hospital, Münster, Germany; 5 Department of Orthopedics and Traumatology, University Hospital, Heidelberg, Germany; 6 Department of General, Visceral and Transplantation Surgery, University Hospital, Heidelberg, Germany; 7 Department of Hematology, Oncology, and Rheumatology, University Hospital, Heidelberg, Germany

**Keywords:** *TERT*, Promoter, Mutation, Soft tissue, Sarcoma, Myxoid liposarcoma, Solitary fibrous tumor

## Abstract

**Background:**

Recently, recurrent point mutations in the telomerase reverse transcriptase (*TERT*) promoter region have been found in many human cancers, leading to a new transcription factor binding site, increased induction of *TERT* and subsequently to telomere maintenance. We determined the prevalence of *TERT* promoter mutations in soft tissue sarcomas of 341 patients comprising 16 entities and in 16 sarcoma cell lines covering 7 different soft tissue sarcoma types.

**Methods:**

The sarcoma tissue samples were collected from the archives of the Institute of Pathology, University of Heidelberg and were composed of 39 myxoid liposarcomas (MLS), 61 dedifferentiated liposarcomas, 15 pleomorphic liposarcomas, 27 leiomyosarcomas, 25 synovial sarcomas (SS), 35 malignant peripheral nerve sheath tumors (MPNST), 40 undifferentiated pleomorphic sarcomas, 17 myxofibrosarcomas, 9 low grade fibromyxoid sarcomas, 10 cases of dermatofibrosarcoma protuberans, 31 solitary fibrous tumors (SFT), 8 extraskeletal myxoid chondrosarcomas, 9 angiosarcomas, 6 alveolar soft part sarcomas, 5 clear cell sarcomas and 4 epithelioid sarcomas. Sarcoma cell lines were obtained from the raising laboratories. A 193 bp fragment of the *TERT* promoter region covering the hot-spot mutations C228T and C250T was amplified, and direct sequencing of the PCR products was performed.

**Results:**

*TERT* promoter mutations were detected in 36/341 sarcomas. They were highly recurrent in MLS (29/39; 74%) and were in the present MLS series not associated with the phenotype (myxoid *vs*. round cell variant), tumor grade, tumor site and patients’ median age or gender. In the remaining cases, *TERT* promoter mutations were found only in 7/302 sarcoma samples and confined to SFTs (4/31; 13%), MPNSTs (2/35; 6%), and SSs (1/25; 4%). Within the collection of sarcoma cell lines examined, *TERT* promoter mutations were detected in two MLS and in one of three MPNST cell lines.

**Conclusions:**

*TERT* promoter mutations are frequent in MLSs including their round cell variants, representing the most prevalent mutation identified in this sarcoma entity to date, and in a minor fraction of SFTs, MPNSTs and SSs. The majority of sarcomas are devoid of *TERT* promoter hotspot mutations. These data suggest that telomere maintenance through increased expression of telomerase plays an important role in the pathogenesis especially of MLS.

## Background

Soft tissue sarcomas (STS) are a highly heterogeneous group of malignant tumors of mesenchymal origin represented by voluntary muscles, fat, and fibrous tissue and their vessels and by convention the peripheral nervous system [1]. STS are relatively rare and constitute approximately 1–2% of all human cancers, but incidence dramatically increases with age [1,2].

Since most patients with STS present with a painless swelling, a delayed diagnosis is common, often with local or distant metastatic spread at the time of diagnosis [2]. The treatment of choice depends on the individual tumor type, grading and staging status. Surgery, among others, is a key element of therapy in sarcomas of adults with the aim of microscopically tumor-negative margins for optimal local control [3]. However, standardized treatment might be insufficient. Under these circumstances, advance in personalized treatment strategies might become important with the goal to individual tumor-targeted therapies.

That is why the biology of STS has intensively been investigated over the last decades with a dramatic increase of knowledge about genetic alterations [4] including aberrant DNA methylation [5]. In general, sarcomas can be classified into two genetic groups: i. sarcomas with specific chromosomal rearrangements on a background of relatively few other chromosomal changes, and ii. sarcomas without specific alterations on a complex background of numerous chromosomal changes [6]. Specific genetic alterations are not only of diagnostic significance, but also might become relevant for tumor-targeted therapies.

Telomere maintenance is an important step during tumorigenesis and confers unlimited proliferative capacity to cancer cells [7]. In principal, two mechanisms can be involved in telomere maintenance: a telomerase dependent mechanism or a non-telomerase dependent mechanism also referred to as Alternative Lengthening of Telomeres (ALT) [7]. The ribonucleoprotein complex telomerase provides the physiological mechanism that maintains telomere length by adding repetitive hexanucleotide repeats with the sequence 5′-TTAGGG-3′ to telomeres. Reactivation of telomerase has been observed in the majority of human cancers [8]. In this context, telomerase reverse transcriptase (*TERT*) serves as the catalytic subunit of the telomerase complex and has been shown to contribute to the immortalization of cancer cells [7]. However, the underlying mechanism of *TERT* reactivation in cancer cells was an unresolved issue [9].

Recently, highly recurrent somatic mutations in the promoter region of the *TERT* gene have been detected [10]. The most frequent mutations were a single cytosine exchange to thymine at chromosome 5 base position 1,295,228 (C228T) or less frequently at base position 1,295,250 (C250T) (-124 and -146 bp from ATG start site, respectively). These *TERT* mutations lead to a new binding motif for E-twenty six/ternary complex factors (Ets/TCF) transcription factors and results in an up to 4-fold increase of *TERT* promoter activity in reporter gene assays [11,12].

First described in melanomas [11,12], *TERT* promoter mutations have subsequently been found in many other human cancer types, with highest frequencies in subtypes of CNS tumors, in a number of malignancies of epithelial origin including bladder carcinomas, thyroid carcinomas, and hepatocellular carcinomas, and in atypical fibroxanthomas and in dermal pleomorphic sarcomas [13-26]. Accordingly, *TERT* promoter mutations belong to the most common somatic genetic lesions in human cancers.

A study by Killela et al. investigated a broad range of human cancers for *TERT* promoter mutations, including soft tissue sarcomas [16]. However, the case number of single STS entities was limited and a number of subtypes were not comprised.

Therefore, the present study was conducted to investigate the prevalence of *TERT* promoter mutations in a comprehensive series of 341 soft tissue tumors comprised of 16 types including rare entities and in 16 cell lines of seven sarcoma types. Further, we looked for associations, if any, with clinicopathological parameters.

## Materials and methods

### Sarcoma samples and clinicopathological characteristics

The sarcoma tissue samples were collected at the Institute of Pathology, University of Heidelberg, and diagnoses were confirmed by three sarcoma pathologists (GM, WH and EW). Diagnoses were based on standard histopathological criteria in conjunction with immunohistological and molecular analysis according to the current WHO classification of tumors [1]. Only samples with at least 80% vital tumor cells were selected for the analysis. The study was approved by the ethics committee, medical faculty of heidelberg University (No. 206/2005, 207/2005). The clinicopathological characteristics are shown in Additional file 1: Table S1. Further molecular and histological data of myxoid liposarcomas are given in Additional file 1: Table S2. The sarcoma cell lines examined, together with references, molecular confirmation and culture conditions are detailed in Additional file 1: Table S3 (according to reference [5]).

### DNA isolation

DNA was extracted from 1 to 3 (depending on the size of the tumor sample) 8 μm thick sections from formalin-fixed and paraffin embedded (FFPE) samples using the Maxwell® 16 FFPE Tissue LEV DNA Purification Kit (Promega, Madison, USA) according to the manufacturer’s instructions. The extracted DNA was quantified with the Nanodrop ND-1000 spectrophotometer (NanoDrop Technologies, Rockland, USA).

### Direct (Sanger) sequencing

A 193 bp fragment of the *TERT* promoter region spanning the hotspot mutations at positions 1,295,228 and 1,295,250 on chromosome 5 was amplified by using GoTaq G2 Hot Start Polymerase (Promega, Madison, USA) and the following primers: hTERT-seq-for 5′-CACCCGTCCTGCCCCTTCACCTT-3′ and hTERT-seq-rev 5′- GGCTTCCCACGTGCGCAGCAGGA-3′. PCR was performed with 100 ng of DNA template in a total volume of 25 μl, and included initial denaturation at 95°C for 120 s, followed by 35 cycles with denaturation at 95°C for 30 s, annealing at 68°C for 30 s, and extension at 72°C for 40 s. In cases where amplification of the large fragment failed, primers hTERT-short-for 5′-CAGCGCTGCCTGAAACTC-3′ and hTERT-short-rev, 5′-GTCCTGCCCCTTCACCTT-3′, which amplify a 163 bp fragment, were applied as described previously [17]. PCR products were purified using USB Exo-SAP-IT (Affymetrix, Cleveland, USA) and direct sequencing of the PCR products was performed for both strands on an ABI 3500 genetic analyzer (Life Technologies, Darmstadt, Germany) using a version 1.1 BigDye Terminator cycle sequencing kit and a BigDye Xterminator purification kit (Life Technologies, Foster City, USA).

### Statistical analysis

Fisher’s exact test was used to examine associations between nominal variables. Student’s t test was used to examine the association between nominal variables and age. Significance was defined as p < 0.05.

## Results

### *TERT* promoter hotspot mutations in soft tissue sarcomas

**Figure 1:**
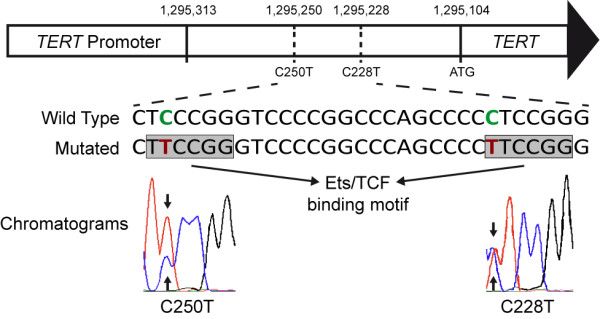
**Schematic figure of the *TERT *promoter region.** Schematic figure of the *TERT* promoter region with nucleotide numbering of the molecular position on chromosome 5, DNA sequence of the mutational hotspot region with a wild type strand and a mutated strand, which shows the nucleotide exchange of cytosine by thymine (depicted in red). Each mutation leads to a new binding motif for E-twenty six/ternary complex factors (Ets/TCF) transcription factors (highlighted by greyish rectangles). Representative sequencing chromatograms show heterozygous C228T/C250T mutations (indicated by arrows).

*TERT* promoter mutations were detected in 36 of 341 sarcoma samples from 341 patients (10.5%; Table 1). The mutations comprised 32 C228T mutations, but only three C250T mutations. They occurred in a mutual exclusive manner with a heterozygous genotype (Figure 1). Mutations were highly recurrent (29/39; 74%) in myxoid liposarcomas (MLS) and were almost exclusively found at position C228T with the exception for one case with a C250T mutation. Remarkably, the 28 MLS carrying a C228T mutation were all positive for the FUS-DDIT3 fusion, while the C250T mutation was found in one of two MLS with an EWSR1-DDIT3 fusion transcript (Additional file 1: Table S2). Looking for associations between the *TERT* promoter mutation status and clinicopathological features of the MLS (Table 2), there were no associations between *TERT* promoter mutational status and phenotype (myxoid *vs.* round cell variant), tumor grading, tumor site and patients’ median age or gender. Solitary fibrous tumors (SFTs) showed *TERT* promoter mutations in four cases (4/31; 13%), which were exclusively located at position C228T. In addition, two malignant peripheral nerve sheath tumors (MPNSTs) harbored a *TERT* promoter mutation (2/35; 6%), one case with a C228T and the other one with a C250T mutation. Finally, a *TERT* promoter mutation at position C228T was found in one of the synovial sarcomas (SSs) examined (1/25; 4%). All other sarcoma types, which comprised 61 dedifferentiated liposarcomas, 15 pleomorphic liposarcomas, 27 leiomyosarcomas, 40 undifferentiated pleomorphic sarcomas, 17 myxofibrosarcomas, 9 low-grade fibromyxoid sarcomas, 10 dermatofibrosarcomata protuberans, 8 extraskeletal myxoid chondrosarcomas, 9 angiosarcomas, 6 alveolar soft part sarcomas, 5 clear cell sarcomas, and 4 epithelioid sarcomas had a wild type genotype at the two *TER*T promoter hotspot loci (Table 1).

**Table 1:** **Prevalence of *TERT *** **promoter hotspot mutations in soft tissue tumors**

**Diagnosis**	**Mut (n)**	**Total (n)**	**Mut (%)**	**C228T (n)**	**C250T (n)**
Myxoid liposarcoma	29	39	74%	28	1
Dedifferentiated liposarcoma	0	61			
Pleomorphic liposarcoma	0	15			
Leiomyosarcoma	0	27			
Synovial sarcoma	1	25	4%		1
Malignant peripheral nerve sheath tumor	2	35	6%	1	1
Undifferentiated pleomorphic sarcoma	0	40			
Myxofibrosarcoma	0	17			
Low grade fibromyxoid sarcoma	0	9			
Dermatofibrosarcoma protuberans	0	10			
Solitary fibrous tumor	4	31	13%	4	
Extraskeletal myxoid chondrosarcoma	0	8			
Angiosarcoma	0	9			
Alveolar soft part tumor	0	6			
Clear cell sarcoma	0	5			
Epithelioid sarcoma	0	4			
	36	341		33	3

**Table 2:** **Correlation between clinicopathological patient characteristics and *TERT *** **promoter genotype in myxoid liposarcomas**

	**Mutant**	**Wild-type**	***P*** **value**
Phenotype (n = 39)			0.2125
Myxoid	23	6	
Round cell	6	4	
Grading (n = 39)			0.6034
G1	3	1	
G2	22	6	
G3	4	3	
Localization (n = 39)			0.1958
Extremity	23	10	
Other	5	0	
Age (years) (n = 39)			0.6748
Mean ± SD	48 ± 3	50 ± 5	
Median (range)	46 (16–84)	43 (36–74)	
Gender (n = 39)			0.6395
Female	9	3	
Male	20	7	

### *TERT* promoter hotspot mutations in soft tissue sarcoma cell lines

We also sequenced 16 sarcoma cell lines for the *TERT* promoter hotspot mutations (Table 3). Mutations were found in both MLS cell lines and in one of three MPNST cell lines. Mutations were restricted to the C228T locus. The genotype at the two hotspot positions of the *TERT* promoter of the remaining cell lines, which included two well-differentiated liposarcomas, one dedifferentiated liposarcomas, one pleomorphic liposarcoma, one liposarcoma not further subtyped, four SSs, and one fibrosarcoma, was wild type.

**Table 3:** **List of soft tissue sarcoma cell lines with the corresponding *TERT *** **promoter mutation status**

**Cell line name**	**Origin**	***TERT*** **promoter**
T449	WDLS	WT
T778	WDLS	WT
FU-DDLS-1	DDLS	WT
MLS402	MLS	C228T
MLS1765	MLS	C228T
LiSa-2	PLS	WT
SW872	LS	WT
1273	SS	WT
HS-SY-II	SS	WT
SYO-1	SS	WT
Fuji	SS	WT
CME	SS	WT
STS26T	MPNST	C228T
ST88-14	MPNST	WT
T265	MPNST	WT
HT1080	FS	WT

*Abbreviations*: *WDLS* well differentiated liposarcoma, *DDLS* dedifferentiated liposarcoma, *MLS* myxoid liposarcoma, *SS* synovial sarcoma, *MPNST* malignant peripheral nerve sheath tumor, *FS* fibrosarcoma, *LS* liposarcoma, *WT* wild type, *C228T* cytosine exchange to thymine at chromosome 5 base position 1,295,228.

## Discussion

Telomere maintenance mechanisms represent a pivotal cornerstone in the development and sustainment of cancer. The recently described mutations in the promoter region of *TERT* provide new evidence for the important role of telomerase reactivation in human cancers. The overall prevalence of *TERT* promoter hotspot mutations was low in the comprehensive series of soft tissue sarcomas examined in this study (36/341; 10.5%). However, the prevalence strongly varied by sarcoma type.

The by far highest mutation rate was found in MLS (29/39; 74%), which represents the most prevalent mutation identified in this sarcoma entity to date, and which corroborates data obtained in a recent study on a smaller series of MLS [16]. In MLS, increased *TERT* transcription [27-29] and telomerase reactivity [28] have been described previously. Costa et al. found telomerase reactivation in 69% of MLS with an additional round cell component (high grade) [28], which is overlapping with the overall *TERT* promoter mutation frequency of 74% (29/39) in our series of MLS. However, in pure MLS without the round cell phenotype (corresponding to low grade), they found telomerase reactivation only in 39% of cases [28]. Likewise, Schneider-Stock et al. detected telomerase activity in 30% of MLS, but elevated *TERT* mRNA levels in a much higher proportion of cases [27,29,30]. Furthermore, intratumoral heterogeneity of *TERT* expression and telomerase activity has been observed in sarcomas, in particular in liposarcomas [31]. Thus, *TERT* mRNA levels are not stringently correlated with telomerase enzyme activity. This might be explained by sufficient regulatory mechanisms of the enzymatic function of telomerase, which still have to be functional in some tumors.

Indeed, regulatory mechanisms of telomerase have already been described at the transcriptional and post-translational level. At the transcriptional level, alternative splicing of *TERT* mRNA itself might not only lead to TERT variants with impaired catalytic functions [32], but also to a variant that acts in a dominant-negative manner on telomerase activity [33]. Furthermore, post-translational modifications of the TERT protein through phosphorylation or ubiquitination have been shown to affect the catalytic activity and stability of TERT [34].

Anyhow, our data suggest that mutation of the *TERT* promoter causes telomerase reactivation in MLS and thereby most probably provides unlimited proliferative potential. This assumption is also underpinned by a reporter gene assay of the two most common mutation variants within the promoter region of *TERT*, namely C228T and C250T, which were shown to lead to an augmented expression of *TERT*[12]. Further, the high prevalence of *TERT* promoter mutations not only in MLS round cell variants but also in MLS with a pure myxoid phenotype, and this irrespective of tumor grading, implies that these mutations act rather as driver than passenger mutations.

*TERT* promoter mutations might also have a diagnostic impact in myxoid sarcomas. Mutations were found neither in dedifferentiated liposarcomasa (DDLS), nor in pleomorphic liposarcomas (PLS), which presented myxoid areas in many cases, and were also not detectable in our series of myxofibrosarcomas, extraskeletal myxoid chondrosarcomas, dermatofibrosarcomata protuberans, and low-grade fibromyxoid sarcomas.

The absence of *TERT* promoter hotspot mutations in our series of DDLS and PLS is in line with previous studies, which largely observed deficient telomerase activity in high-grade liposarcomas. Instead, high-grade liposarcomas often use the ALT mechanism [28,35,36]. ALT overcomes telomere attrition through homologous recombination of telomeric DNA and characteristically presents with a pattern of telomere lengths that range from very short to abnormally long. This telomere pattern is clearly different compared to tumors with telomerase reactivation, where telomere length is found almost equal [36].It has been shown that ALT-positive liposarcomas have a notably worse outcome, and may imply a more favorable prognosis for *TERT* promoter mutated liposarcomas [28,37,38]. However, differences in patients outcome might be dedicated to the fact that telomere maintenance via ALT is more often applied by tumors with complex karyotypes or with a higher level of genomic instability [39,40], whereas sarcomas characterized by type specific translocations rather use telomerase reactivation for telomere maintenance [39,41]. According to our data, this concept holds true for the group of liposarcomas. MLS are characterized by a translocation that fuses the *DDIT3* (*CHOP*) gene on chromosome 12q13 with the *FUS* (*TLS*) gene on chromosome 16p11 in approximately 90% of cases, or the *DDIT3* (*CHOP*) with the *EWSR1* on chromosome 22q12 in the remaining cases [42]. By contrast, DDLS typically have complex karyotypic aberrations with amplification of the chromosome 12 subregion q13-15, which includes the *murine double minutes* (*MDM2*) and *cyclin dependent kinase-4* (*CDK4*) genes among others [43,44]. PLS are characterized by highly complex karyotypes [45]. The highest prevalence of ALT has been observed in DDLS and PLS, which typically have an aggressive biological behavior [28,37]. However, *TERT* promoter mutated MLS may undergo malignant progression to the round cell variant and then present with a similar biological behavior like ALT-positive PLS [46]. Another fact that challenges this concept is that patients suffering from ALT-positive glioblastoma have a more favorable clinical course compared to ALT-negative counterparts [47,48]. Thus, the unfavorable prognosis in ALT-positive liposarcomas is probably derived from the mutational signature in these tumors rather than dependent on the mechanism of telomere maintenance, and thus may considerably differ between different tumor entities.

The second most common rate of *TERT* promoter mutations was observed in SFT with a frequency of 13%, which is concordant to data on a smaller series of SFTs [16]. However, *TERT* promoter mutation might be dependent on the anatomic site of presentation, since cranial SFTs and hemangiopericytomas, which are now considered to belong to the SFT family from a genetic perspective [49], have a slightly higher mutation frequency (11/43; 26%) [17].

In MPNSTs, *TERT* promoter mutations were found in a small fraction of tumors (2/35; 6%), which is slightly below the mutation frequency previously reported (2/12; 17%) [17]. These data might suggest a minor significance in this tumor entity. On the other hand, one out of three MPNST cell lines was revealed with a *TERT* promoter mutation, which supports the assumption that telomerase reactivation by *TERT* promoter mutations might contribute to immortalization of at least a small proportion of MPNSTs. Interestingly, a previous study that focused on telomerase activity in MPNSTs found telomerase reactivation in 14 of 23 (61%) MPNSTs [50]. Compared with histological grade, telomerase activity was completely restricted to high grade MPNSTs (14/17; 82%) in that study. Indeed, the two MPNSTs with *TERT* promoter mutation described here presented with typical histological features of high-grade MPNSTs [51]. Moreover, in another study on 57 MPNST samples telomerase activity proved to be significantly associated with disease-specific mortality during 5 years of follow-up [52].

Another notable observation is the sporadic occurrence of *TERT* promoter mutations in SSs. This tumor typically applies telomerase reactivation for telomere maintenance [53], which is in concordance with our own observations (data not shown). However, like in MPNSTs, *TERT* promoter hotspot mutations just play a minor role in SSs with merely a single mutated case in our series (1/25; 4%). Thus, the low mutation frequencies in MPNSTs and SSs suggest that a so far unknown mechanism beside the *TERT* promoter hotspot mutations may exist that provides telomerase reactivation.

Explanations for telomerase maintenance get complicated by the observation that a considerable fraction of STS do neither apply telomerase activation nor the ALT mechanism that is so far known, or even may be equipped with both mechanisms [7,36]. Further studies concerning molecular alterations in STS will in particular draw more attention to the non-coding genomic regions and hopefully elucidate the remaining unanswered questions, which mechanisms these tumors exploit to prevent telomere attrition.

## Conclusion

We determined the prevalence of *TERT* promoter hotspot mutations in STS. Despite the overall low prevalence in this tumor group, *TERT* promoter mutations revealed to be a highly recurrent event in MLS and currently represent the most prevalent mutation identified in this sarcoma entity (74%). Forthcoming studies will be needed to determine whether the *TERT* promoter mutational status could be of clinical relevance, especially in advanced MLS. Additionally, *TERT* promoter mutations were also found in a subset of SFTs (13%), and in a number of MPNSTs (6%) and SSs (4%). Given the relative frequency of telomerase activation reported in MPNSTs and in SSs, the low *TERT* promoter mutation rate in these sarcoma types implies that a so far unknown mechanism, different from the presently known *TERT* promoter hotspot mutations, provides telomerase reactivation in these sarcoma entities.

## Competing interests

The authors declare that they have no competing interests.

## Authors’ contributions

CK and MR contributed equally to this work. CK, MR, WH, EW, TS, GE, PS, AvD and GM performed data analyses. WH, EW and GM carried out the histological review of cases. CK, MR, NW and RP performed molecular analyses. AU, ERK, BL, IA, PS and GM collected cases. CK and GM conceived and designed the study, and prepared the initial manuscript. GM supervised the project. All authors contributed to the final manuscript. All authors read and approved the final manuscript.

## Supplementary Material

Additional file 1: Table S1 Clinicopathological patients’ characteristics. Internal identifier, diagnosis, patients’ age at surgery, gender, tumor localization, presence/absence of a fusion transcript, and *TERT* promoter mutational status with nucleotide exchange, are indicated for all cases. Abbreviations: UPS = undifferentiated pleomorphic sarcoma; DDLS = dedifferentiated liposarcoma; PLS = pleomorphic liposarcoma; MLS = myxoid liposarcoma; LMS = leiomyosarcoma; SS = synovial sarcoma; MFS = myxofibrosarcoma; MPNST = malignant nerve sheath tumor; EMCS = extraskeletal myxoid chondrosarcoma; SFT = solitary fibrous tumors; ASPS = alveolar soft part sarcoma; CCS = clear cell sarcoma; EPS = epithelioid sarcoma; DFSP = dermatofibrosarcoma protuberans; LGFMS = low-grade fibromyxoid sarcoma; AS = angiosarcoma. **Additional file 1: Table S2**. Molecular and histological features of the myxoid liposarcomas. Internal identifier, patients’ age at surgery, gender, tumor localization, and *TERT* promoter mutational status with nucleotide exchange together with information about the histological phenotype, tumor grading and presence/absence of gene fusions (*FUS*-*DDIT3*; *EWSR1*-*DDIT3*) are given for myxoid liposarcomas. Abbreviations: MLS = myxoid liposarcoma; MYX = myxoid; RC = round cell; wt = wild type; mut = mutated; DDIT3 split = FISH probe indicates interrupted gene; na = not available; nd = not determinable. **Additional file 1: Table S3**. Soft tissue sarcoma cell lines. Cell line name, tissue of origin (soft tissue sarcoma subtype), molecular confirmation, culture conditions and source/reference is given for each cell line tested for *TERT* promoter mutations. Abbreviations: FCS = fetal calf serum; PS = penicillin and streptomycin; PMID = PubMed identifier. Click here for file
